# From Learning *Healthcare* Systems to Learning *Health* Systems

**DOI:** 10.1002/lrh2.10216

**Published:** 2020-01-24

**Authors:** Aziz Sheikh

**Affiliations:** ^1^ Primary Care Research and Development, Usher Institute The University of Edinburgh Edinburgh UK

**Keywords:** data science, health policy, population health

1

There is growing national and international interest in creating learning healthcare systems; this drive is stimulated by the (then) Institute of Medicine's (now National Academy of Medicine) 2007 seminal report on the Learning Healthcare System.^1^ In this report, the Institute of Medicine urged for the creation of a model of care “…that is designed to generate and apply the best evidence for the collaborative healthcare choices of each patient and provider; to drive the process of discovery as a natural outgrowth of patient care; and to ensure innovation, quality, safety, and value in healthcare.” Over the ensuing 12 years, this bold idea has catalyzed considerable scientific, clinical, and policy interest in finding ways to converge the processes of knowledge generation and practice improvement and through doing so, simultaneously improving healthcare delivery and personalization of care^2^ whilst also containing healthcare expenditure.^3^ Whilst this expanding interest is welcome in many respects, I believe the emphasis on Learning *Healthcare* Systems is misplaced. My contention is that we need to focus on the creation of Learning *Health* Systems. There are two key reasons why I believe we need to shift our focus, which I summarize below.

First, there is increasing recognition that health—the improvement of which is our ultimate goal—is only poorly correlated with healthcare provision or expenditure.^4^ Estimates suggest that healthcare is responsible for only 15% to 40% of population health outcomes.^5^ Far more important at a population level are the wider determinants of health, the majority of which fall outside the ambit of traditional healthcare provision. For example, housing can impact on mental health,^6^ and road planning can have a major impact on the risk of road traffic accidents.^7^ If we want to impact on these and related health outcomes such as reducing the risks of morbidity and mortality from firearms, we need to look well beyond healthcare. The undue focus on healthcare systems limits the opportunity to understand and impact on these and numerous other societal determinants of health.

Second, related concern is that healthcare is, in many countries, delivered through modest sized autonomous or semi‐autonomous institutions thereby limiting our ability to think at scale—by which I mean at state, national, regional, or indeed global levels. So much of health is either won or lost depending on the policy decisions that are made by state, federal, or intergovernmental bodies—for example, taxation on sugary drinks,^8^ age limits for the consumption of alcohol,^9^ and legislation on smoke‐free public places.^10^ It is crucial that we also develop evidence informed approaches to such crucial macro‐level policy decisions when considering how best to improve health.^11^ In order to do so effectively, it is important to expand the stakeholder base to, in addition to healthcare providers, include, for example, policymakers, public health/population health practitioners, and social workers and in the case of the firearms example cited above other agencies such as law enforcement.

It is helpful at this point to reflect on why the original focus has been on Learning *Healthcare* Systems, often conceptualized more narrowly still as synonymous with Learning *Hospital* Systems. This reflects the fact that the idea of Learning Healthcare Systems originated in the United States, which at the time had (and retains) a focus on hospitals as the main vehicle for delivering health. In contrast to a number of other high‐income countries, ambulatory, community, and public health models of care delivery are less well developed in the United States. For example, in the United Kingdom, over 90% of all healthcare interactions now take place in community‐based settings—mainly with general practitioners (family physicians) and their increasingly multidisciplinary teams.^12^ Had this idea been given birth to elsewhere, there may well have been less of a focus on hospital settings.^13^ The Institute of Medicine has signalled a shift in this direction through, for example, its report on *Digital Infrastructure for the Learning Health System*, but there is still a long way to go if this is to be made real.^14^

Looking ahead, we need to collectively expand the focus from Learning *Healthcare* Systems to Learning *Healt*h Systems. Taking this simple but profound conceptual leap will enable us collectively to develop more holistic approaches to the data‐enabled ongoing transformation of health outcomes.^15^ Thankfully, such a move will not require any change to the terminology of The Learning Cycle^16^ or indeed the name of this Journal!

## CONFLICT OF INTERESTS

Aziz Sheikh is Co‐Director of Harvard Medical School's Safety Quality and Informatics Leadership program, Co‐Director of the NHS Digital Academy and was Chair of the World Innovation Summit for Health's (WISH) Forum on Harnessing Data Science and AI in Healthcare.
